# How Different Physical Qualities Influence Repeated Sprint Ability Tests in Elite Youth Soccer Players?

**DOI:** 10.5114/jhk/203832

**Published:** 2025-09-23

**Authors:** Alejandro Sal-de-Rellán, Mehdi Ben Brahim, Víctor Martín, Najet Zidi, Ariadna Hernaiz-Sánchez

**Affiliations:** 1 Department of Education and Educational Innovation, Faculty of Law, Education and Humanities, Universidad Europea de Madrid, Madrid, Spain.; 2 Health and Physical Education Department, Prince Sultan University, Riyadh, Kingdom of Saudi Arabia.; 3 Faculty of Health Sciences, Universidad Isabel I, Burgos, Spain.

**Keywords:** RSA, physical qualities, team sport games, sprinting, football

## Abstract

The aim of this study was to examine the relationships between different repeated sprint ability (RSA) tests and to determine whether all RSA tests were related to the same physical qualities. Twenty-five young elite soccer players (age: 17.1 ± 0.9 years; body height: 172.5 ± 4.6 cm; body mass: 68.1 ± 3.9 kg) participated in this study. Participants completed five RSA tests (i.e., RSA 20 + 20, RSA-linear, RSARANDOM, RSA 15 + 15, and RSA-Curve), a linear sprint test (i.e., a 30-m linear sprint with split times recorded at the 5^th^ and the 10^th^ m ), a curve sprint test (CV_Best and CV_Worst), a change of direction (COD) test, and vertical jump tests (i.e., SJ, CMJ and DJ tests) during a training camp. The main results showed moderate relationships between RSA-RANDOM and RSA 20 + 20 (r = 0.410; p < 0.05) and between RSA-Linear and RSA 15 + 15 (r = 0.475; p < 0.05). In addition, negative relationships were observed between RSA-Curve and the other RSA tests. Additionally, the RSA-Linear test obtained moderate relationships with a 10-m sprint (r = 0.485; p < 0.05), CV_Best (r = 0.484; p < 0.05), CV_Worst (r = 0.410; p < 0.05) and the CMJ (r = 0.403; p < 0.05), and the RSA-RANDOM test with COD (RSA_total_: r = 0.414; p < 0.05; RSA_best_: r = 0.489; p < 0.05). These findings may help coaches and sports scientists optimize training and performance assessment by identifying the most suitable RSA test for specific variables. In addition, training focused on improving different physical qualities can positively impact RSA performance.

## Introduction

Soccer is an intermittent team sport that requires a variety of high-intensity actions, including accelerations, decelerations, changes of direction, jumping, and kicking the ball, interspersed with intervals of low intensity (Nobari et al., 2023b; Pons et al., 2021). On average, a soccer player covers a total distance of 10–12 km per match (Bangsbo et al., 2006). Additionally, players usually cover approximately 418–568 m per match running at a very high-speed (i.e., 21–24 km•h^−1^) (Rey et al., 2023) and perform about 100–150 accelerations (Varley and Aughey, 2013). Moreover, soccer players have to repeatedly perform maximal or near-maximal short-duration sprints (1–7 s) with brief recovery intervals (Girard et al., 2011; Kyles et al., 2023), thus repeated sprint ability (RSA) is a key factor for success (Sal de Rellán-Guerra et al., 2019).

Various fitness assessments have been employed to collect objective data on the athletes' fitness levels, to develop appropriate short- and long-term training regimens, and to offer them motivation and feedback (Nobari et al., 2023a; Rampinini et al., 2007; Svensson and Drust, 2005). At first, “traditional” RSA tests (i.e., straight line) included different distances (15 to 40 m), a different number of sprints (i.e., 5 to 15), and different recovery protocols (i.e., 20 to 40 s, active or passive). Subsequently, changes of direction began to be included in these tests, with a 180º change of direction (i.e., a shuttle run test) being the most commonly used (Impellizzeri et al., 2008). Both tests have been able to discriminate between playing standards (Rampinini et al., 2007), and performance in these tests is strongly correlated with distance covered at very high intensity in a match (Rampinini et al., 2007). However, these tests have not been able to include the unexpected occurrences during matches (Carling et al., 2012). In this regard, Martin et al. (2018) presented an RSA test (6 sets of 20 m [10 + 10 m, COD or linear]) which included decision-making (RSA-RANDOM test). Those authors demonstrated that the RSA-RANDOM test could reliably assess RSA performance in youth soccer players. For this reason, more soccer field performance tests have been developed in the last few years to better simulate soccer matches (Di Mascio et al., 2015; Fílter et al., 2020a; Martin et al., 2018).

Due to the importance of RSA in soccer (Bishop et al., 2011; Rampinini et al., 2007), and the limited time available for its assessment, it would be interesting to know whether RSA tests are related to other physical traits. For example, research on young soccer players delved into the relationship between RSA (8 sets of 30 m with a rest of 25 s) and several performance variables associated with aerobic and anaerobic performance in soccer (Rodríguez-Fernández et al., 2019). It was concluded that RSA was more strongly correlated with aerobic variables that incorporated neuromechanical components with VO_2max_. It was also observed that adaptations varied based on the players’ initial aerobic fitness levels (i.e., low vs. high), with those possessing high aerobic fitness deriving greater benefits from a repeated sprint program (Sanchez-Sanchez et al., 2019). Another work revealed a stronger relationship between RSA performance and fitness determinants such as acceleration, agility, explosive leg power, and aerobic conditioning (Spencer et al., 2011). Results showed significant relationships between the total time obtained in the RSA test (RSA_total_) compared to the 15-m sprint, the 15-m agility run, and the countermovement jump (CMJ) in U18 soccer players. This suggests that stabilization of overall physical performance is evident in this age group, and good fitness performance in RSA will also translate into other physical abilities (e.g., jumping and sprinting).

RSA testing protocols are highly variable and different (i.e., sprint distance, the number of sprints, the type and time of recovery, the number and the type of direction changes, inclusion of uncertainty); thus, a player's performance in an RSA test may vary depending on the characteristics of the particular test. In order to optimize test selection and solve the issue of little time availability in soccer, it would be necessary to evaluate the different determinants of RSA tests. It has been shown that performance in the first sprints (1–3) is related to both the player's strength and sprinting ability, while from the fourth sprint onwards, the participation of aerobic capacity increases (López-Segovia et al., 2015). The type and time of recovery are also very important. Shorter recovery times between sprints cause more lactate to build up in the blood (Glaister, 2005), while passive recovery leads to better performance in the average time that an RSA test takes (Buchheit et al., 2009).

To the authors’ knowledge, no study has compared youth soccer players’ performance in different types of RSA tests (linear, change of direction, uncertainty, or curved) to determine whether these are based on the same physical qualities. For this reason, the findings of this study could be of great interest for strength and conditioning coaches of professional soccer teams in order to improve physical performance and optimize time when selecting the most appropriate RSA test. Therefore, the main objective of this research was to establish the relationship between physical performances in different RSA tests in elite youth soccer players. Furthermore, in order to analyse the relationship between different physical qualities and RSA, various fitness tests were included. Given the above (Impellizzeri et al., 2008; Martin et al., 2018; Rodríguez-Fernández et al., 2019), it was hypothesized that due to the specific characteristics of each RSA test considered, these would not be related to the same physical qualities.

## Methods

### Participants

Twenty-five elite youth male soccer players (age: 17.1 ± 0.9 years; body height: 172.5 ± 4.6 cm; body mass: 68.1 ± 3.9 kg; systematic practice: 7.84 ± 0.98 years) from the Tunisian national team volunteered to participate in this study. Participants were selected using a non- probability convenience sampling method, as the study focused on a specific group of athletes regularly competing in national and international tournaments. Players were classified as tier 4 according to the Participant Classification Framework (McKay et al., 2022). The field players were eight central-defenders, five central-midfielders, five wide-midfielders and seven centre-forwards. The inclusion criteria considered were: a) having completed all assessment sessions, b) participation in ≥ 90 of the total number of training sessions, c) no injuries in the eight weeks prior to the study, d) being an outfield soccer player. Before signing the consent form for testing, participants were informed of the experimental procedures, potential risks, and benefits associated with the investigation. The study was approved by the ethics committee of Universidad Isabel I, Burgos, Spain (protocol code: Uil-PI104; approval date: 16 July 2024) and performed in accordance with the Declaration of Helsinki (2013).

### Design and Procedures

**Figure 1:**
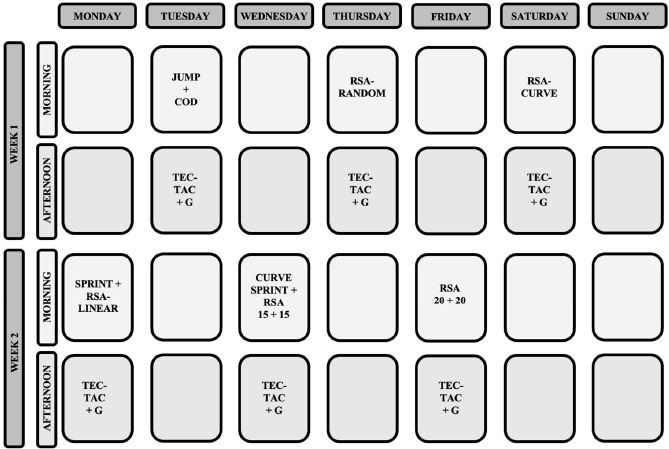
Periodization of training during the research period. TEC/TAC + G = Technical and tactical training + game; Jump = CMJ, SJ and DJ tests; COD = change of direction test; RSA-RANDOM = 6 sets of 20 m (10+10 m, COD) with a rest of 20 s; RSA-Curve = 6 x 17-m (3 for each side) curvilinear sprints with a rest of 20 s; Sprint = linear sprint test; RSA-Linear = 8 sets of 30 m with a rest of 25 s; Curve sprint = curve sprint test; RSA 15 + 15 = 5 x 30-m shuttle sprints (15 m + 15 m) with a rest of 30 s; RSA 20 + 20 = 6 sets of 20+20 m with a rest of 20 s

The present study employed a quasi-experimental longitudinal design. The research was carried out during the season, when players were selected by the national team for a training camp (four weeks). During the first two weeks, players were familiarized with the testing procedures. After familiarization, players performed six evaluation and six training sessions (Figure 1) with the aim of preparing for international competitions. The training sessions included a warm-up (i.e., activation with injury prevention drills), soccer-specific strength and conditioning (i.e., small-sided games, 3 vs. 3 to 6 vs. 6), and technical-tactical content (i.e., 10 vs. 10 with goalkeepers). The session time was distributed as follows: 20%, 50% and 30%, respectively. These sessions were 70–90 min long, while the evaluation sessions lasted for 30–45 min. All evaluation sessions were conducted outdoors, on natural grass, consistently scheduled from 9:30 to 10:30 AM, and under similar environmental conditions (23–25ºC), with the same sports clothes. The evaluation tests were performed every 48 h during the training camp. Players were advised to adhere to their nutritional regimens by abstaining from caffeine-laden beverages, such as coffee, prior to evaluations. A strength and conditioning specialist supervised all the tests, providing verbal encouragement during execution. Before assessment sessions, a general and specific warm-up routine was executed, involving 3-min jogging, followed by 5-min of dynamic and ballistic stretching, and 7-min of progressive sprints and accelerations (Ben Brahim et al., 2023). On the first day of the evaluation, vertical jump and change of direction (COD) tests were conducted. The second day included the RSA-RANDOM test, followed by the RSA-Curve test on the third day. On the fourth day, a linear sprint test and the RSA-Linear test were performed. The fifth day included a curve sprint test and the RSA 15 + 15 test, while the evaluation concluded on the sixth day with the RSA 20 + 20 test.

### RSA Tests

Players performed different RSA tests. Each sprint started from a standing position, with their front foot planted 0.5 m behind the timing gate (WittySEM, Microgate®, Bolzano, Italy). Photocells were positioned 90 cm above the ground (at hip height, approximately), and participants began each trial in a staggered stance with their preferred leg forward. The variables analyzed in the different RSA tests were the following: RSA_total_ = sum of the time of the nº of sprints performed in the test; RSA_best_ = the time of the fastest sprint in the test; %Change = the percentage of change calculated as ([last sprint − first sprint]/first sprint)*100 (Pyne et al., 2008), and S_dec_ = decrement of sprint performance calculated as ([RSA_total_/RSA_best_ x nº of sprints in the test]*100) −100 (Mujika et al., 2009). The tests used were as follows: (1) RSA 20 + 20 test: the test consisted of 6 sets of 2 x 20-m sprints with 180º COD and 20 s of passive recovery in between (Impellizzeri et al., 2008); (2) RSA-Linear test: an RSA test that had 8 maximal 30-m sprints. There were 25 s of active recovery between each sprint. The participants lined up 0.5-m behind the first photocell about two seconds before each sprint (Rodríguez-Fernández et al., 2019); (3) RSA-RANDOM test: the test comprised 6 x 20-m sprints, each consisting of two 10-m segments, with a 20-s active recovery period during the return to the starting position. Each sprint consisted of a 10-m linear sprint followed by a 10-m sprint with three possible directional options: a linear sprint, a 45º change of direction to the right, or a 45º change of direction to the left, as indicated by a traffic light positioned after the initial 10-m linear sprint (Martin et al., 2018); (4) RSA 15 + 15 test: the test consisted of 5 sets of 2 x 15-m sprints with 180º COD and 30 s of passive recovery between bouts (Raya-González et al., 2021); (5) RSA-Curve test: the test consisted of performing 6 curvilinear sprints of 17 m (3 for each side) with 20 s of passive recovery between each bout. This RSA test was an adaptation of the curvilinear sprint test (Fílter et al., 2020a).

### Linear Sprint Test

Players started from a standing position, 0.5 m behind the first timing gate (WittySEM, Microgate®, Bolzano, Italy), and next sprinted at maximum velocity towards the subsequent timing gate. Participants were instructed to perform two maximum sprints of 30 m, with split times recorded at the 5^th^ m and the 10^th^ m, and were permitted a 2-min passive recovery between trials. The fastest time was registered for further analysis.

### Curve Sprint Test

The semi-circle of the goalkeeper area on an official soccer pitch served as the trajectory of the curve sprint test, which was standardized as follows: a radius of 9.15 m from the penalty spot, a straight-line distance of 14.6 m from the initial to the final point, an amplitude angle of 105.84º from the penalty spot, and a total distance of 17 m (Fílter et al., 2020a). Two pairs of photocells (WittySEM, Microgate ®, Bolzano, Italy) were placed at both the start and the end of the curved trajectory. Participants performed two sprints on each side, with a two-minute rest interval between attempts. Both the best and worst performances (s) were selected for subsequent analysis.

### COD Test

Players conducted the 505 test to evaluate COD. Participants began 10 m from the start line, sprinted across the start/finish line, executed a 180° pivot at the 15-m mark, and then returned as quickly as possible across the start/finish line (Dos’Santos et al., 2020). The fastest trial time was recorded for further analysis.

### Vertical Jump Tests

Testing involved the performance of maximal squat jumps (SJs), countermovement jumps (CMJs), and drop jumps (DJs). All jumps were executed on an OptoJump platform (Optojump, Microgate, Bolzano, Italy). Take-off and landing were standardized to achieve full extension of the knee and the ankle at the same location. Participants were directed to optimize jump height while reducing ground contact time during the DJ following a 20-cm drop from a box (Ramírez-Campillo et al., 2015). Each test was performed twice, separated by 60 s of passive recovery, and the best result (cm) was considered for subsequent analysis.

### Statistical Analysis

Descriptive data are presented as the mean ± standard deviation (SD). The Kolmogorov-Smirnov and Levene’s tests were used to verify the normality of the data distribution and the homogeneity of variances. Since all variables had a normal distribution, the Pearson’s correlation coefficient (r) with a 95% confidence interval (CI) was used to examine the relationship among RSA tests. The following scale of magnitudes was followed to interpret the correlation coefficients: < 0.1, trivial; 0.1–0.3, small; 0.3–0.5, moderate; 0.5–0.7, large; 0.7–0.9, very large; and > 0.9, nearly perfect (Hopkins et al., 2009). If 95% confidence limits overlapped small positive and negative values, the magnitude was deemed unclear; otherwise, the magnitude was deemed to be that observed. The significance level was established at *p* < 0.05, and the IBM SPSS Statistics software for Mac, version 25.0 (IBM Corp., Armonk, NY, USA), was used to perform the statistical analysis.

## Results

The relationships among RSA tests are presented in Table 1. Moderate relationships in RSA_total_ were observed between the RSA-RANDOM test and the RSA 20 + 20 test (r = 0.410; *p* < 0.05), while negative relationships were observed between the RSA-Curve test and the RSA-linear test (r = −0.453; *p* < 0.05), the RSA-RANDOM test (r = −0.503; *p* < 0.05), and the RSA 15 + 15 test (r = −0.431; *p* < 0.05), respectively. Furthermore, moderate relationships were found between the RSA 15 + 15 and RSA-linear test (r = 0.475; *p* < 0.05) in RSA_best_. Finally, negative relationships in %Change were observed between the RSA-Curve test and the RSA-RANDOM test (r = −0.479; *p* < 0.05).

**Table 1:** Relationships among RSA tests.

Variable	Test	RSA 20 + 20	RSA-Linear	RSA-RANDOM	RSA 15 + 15
**RSA_total_**	**RSA-Linear**	**−0.211** **(−0.56 to 0.20)**	**-**		
	**RSA-RANDOM**	0.410* **(0.02 to 0.70)**	**0.188** **(−0.22 to 0.54)**	**-**	
	**RSA** **15 + 15**	**−0.102** **(−0.48 to 0.31)**	**−0.193** **(−0.55 to 0.22)**	**−0.374** **(−0.67 to 0.03)**	**-**
	**RSA-Curve**	**−0.222** **(−0.57 to 0.19)**	−0.453* **(−0.72 to −0.07)**	−0.503* **(−0.75 to −0.14)**	−0.431* **(−0.71 to −0.04)**
**RSA_best_**	**RSA-Linear**	**0.014** **(−0.38 to 0.41)**	**-**		
	**RSA-RANDOM**	**0.260** **(−0.15 to 0.59)**	**0.350** **(−0.05 to 0.66)**	**-**	
	**RSA** **15 + 15**	**0.028** **(−0.37 to 0.42)**	0.475* **(0.10 to 0.73)**	**−0.063** **(−0.45 to 0.34)**	**-**
	**RSA-Curve**	**0.038** **(−0.36 to 0.43)**	**−0.277** **(−0.61 to 0.13)**	**−0.277** **(−0.61 to 0.13)**	**0.197** **(−0.22 to 0.55)**
**%Change**	**RSA-Linear**	**0.115** **(−0.29 to 0.49)**	**-**		
	**RSA-RANDOM**	**0.175** **(−0.24 to 0.53)**	**−0.094** **(−0.47 to 0.31)**	**-**	
	**RSA** **15 + 15**	**0.160** **(−0.25 to 0.52)**	**−0.106** **(−0.48 to 0.30)**	**0.161** **(−0.25 to 0.52)**	**-**
	**RSA-Curve**	**−0.252** **(−0.59 to 0.16)**	**−0.052** **(−0.44 to 0.35)**	−0.479* **(−0.74 to −0.10)**	**−0.034** **(−0.42 to 0.37)**
**Sdec**	**RSA-Linear**	**−0.037** **(−0.43 to 0.36)**	**-**		
	**RSA-RANDOM**	**−0.134** **(−0.50 to 0.28)**	**0.207** **(−0.21 to 0.56)**	**-**	
	**RSA** **15 + 15**	**0.081** **(−0.33 to 0.46)**	**0.222** **(−0.19 to 0.57)**	**−0.168** **(−0.53 to 0.24)**	**-**
	**RSA-Curve**	**−0.012** **(−0.41 to 0.39)**	**0.007** **(−0.39 to 0.40)**	**0.130** **(−0.28 to 0.50)**	**0.072** **(−0.33 to 0.46)**

RSA 20 + 20 = 6 sets of 20+20 m with a rest of 20 s; RSA-Linear = 8 sets of 30 m with a rest of 25 s; RSA-RANDOM = 6 sets of 20 m (10+10 m, COD) with a rest of 20 s; RSA 15 + 15 = 5 x 30-m shuttle sprints (15 m + 15 m) with a rest of 30 s; RSA-Curve = 6 x 17-m (3 for each side) curvilinear sprints with a rest of 20 s; RSA_total_ = total time to cover all the sprints; RSA_best_ = the best time obtained in a single sprint; %Change = percentage of change between the first and the last sprint; Sdec = fatigue index. ^⁎^ p < 0.05

Relationships between the RSA tests and fitness tests are shown in Table 2. Negative relationships were observed between the RSA 20 + 20 test (RSA_best_) and CV_Worst (r = −0.435; *p* < 0.05). Moderate relationships were observed between the RSA-Linear test (RSA_best_) and the 10-m sprint (r = 0.485; *p* < 0.05); as well as between the RSA-Linear test (%Change) and CV_Best (r = 0.484; *p* < 0.05), CV_Worst (r = 0.410; *p* < 0.05), and the CMJ (r = 0.403; *p* < 0.05), respectively. Moreover, moderate relationships were observed between the RSA-RANDOM test (RSA_total_ and RSA_best_) and the COD test (r = 0.414; *p* < 0.05; r = 0.489; *p* < 0.05).

**Table 2:** Relationships between each RSA test and fitness tests.

Test	Variable	5 m	10 m	30 m	CV_Best	CV_Worst	COD	SJ	CMJ	DJ
RSA 20 + 20	RSA_total_	0.311 (−0.10 to 0.63)	0.003 (−0.39 to 0.40)	0.278 (−0.13 to 0.61)	−0.255 (−0.59 to 0.16)	−0.234 (−0.58 to 0.19)	0.204 (−0.21 to 0.55)	−0.268 (−0.60 to 0.14)	−0.374 (−0.67 to 0.03)	−0.388 (−0.68 to 0.01)
	RSA_best_	0.150 (−0.26 to 0.52)	0.117 (−0.29 to 0.49)	0.096 (−0.31 to 0.47)	−0.159 (−0.52 to 0.25)	−0.435* (−0.71 to −0.05)	0.098 (−0.31 to 0.48)	−0.281 (−0.61 to 0.13)	−0.323 (−0.64 to 0.08)	−0.315 (−0.63 to 0.09)
	%Change	0.035 (−0.37 to 0.42)	0.128 (-0.28 to 0.50)	−0.279 (−0.61 to 0.13)	0.110 (−0.30 to 0.48)	−0.023 (−0.41 to 0.38)	0.288 (−0.12 to 0.61)	−0.018 (−0.41 to 0.38)	0.175 (−0.24 to 0.53)	0.121 (−0.29 to 0.49)
	Sdec	0.319 (−0.09 to 0.63)	−0.164 (−0.53 to 0.25)	0.330 (−0.07 to 0.64)	−0.214 (−0.56 to 0.20)	0.214 (−0.20 to 0.56)	0.200 (−0.21 to 0.55)	−0.066 (−0.45 to 0.34)	−0.181 (−0.54 to 0.23)	−0.216 (−0.56 to 0.20)
RSA-Linear	RSA_total_	−0.129 (−0.50 to 0.28)	0.261 (−0.15 to 0.60)	0.078 (−0.33 to 0.46)	0.222 (−0.19 to 0.57)	0.027 (−0.37 to 0.42)	0.134 (−0.28 to 0.50)	0.093 (−0.31 to 0.47)	0.199 (−0.21 to 0.55)	−0.064 (−045 to 0.34)
	RSA_best_	−0.080 (−0.46 to 0.37)	0.485* (0.11 to 0.74)	0.329 (−0.08 to 0.64)	0.057 (−0.35 to 0.44)	−0.138 (−0.51 to 0.27)	0.223 (−0.19 to 0.57)	0.066 (−0.34 to 0.45)	−0.001 (−0.40 to 0.39)	−0.217 (−0.56 to 0.20)
	%Change	−0.033 (−0.42 to 0.37)	0.374 (−0.03 to 0.67)	−0.294 (−0.62 to 0.11)	0.484* (0.11 to 0.57)	0.410* (0.02 to 0.69)	−0.033 (−0.42 to 0.37)	0.323 (−0.08 to 0.64)	0.403* (0.01 to 0.39)	0.384 (−0.01 to 0.68)
	Sdec	−0.074 (−0.46 to 0.33)	−0.339 (−0.65 to 0.07)	−0.377 (−0.67 to 0.02)	0.221 (−0.19 to 0.57)	0.229 (−0.18 to 0.57)	−0.138 (−0.51 to 0.27)	0.030 (−0.37 to 0.42)	0.273 (−0.14 to 0.60)	0.222 (−0.19 to 0.57)
RSA -RANDOM	RSA_total_	0.215 (−0.20 to 0.56)	0.283 (−0.13 to 0.62)	0.351 (−0.05 to 0.66)	0.041 (−0.36 to 0.43)	0.271 (−0.14 to 0.60)	0.414* (0.02 to 0.70)	0.133 (−0.28 to 0.50)	0.037 (−0.36 to 0.43)	−0.128 (−0.50 to 0.28)
	RSA_best_	0.311 (−0.10 to 0.63)	0.291 (−0.12 to 0.62)	0.389 (−0.01 to 0.68)	−0.026 (−0.42 to 0.37)	0.178 (−0.23 to 0.54)	0.489* (0.12 to 0.74)	0.015 (−0.38 to 0.41)	−0.075 (−0.46 to 0.33)	−0.183 (−0.54 to 0.23)
	%Change	0.118 (−0.29 to 0.49)	0.081 (−0.32 to 0.46)	0.137 (−0.27 to 0.51)	−0.169 (−0.53 to 0.24)	−0.102 (−0.48 to 0.31)	−0.197 (−0.55 to 0.22)	−0.058 (−0.44 to 0.35)	0.175 (−0.24 to 0.53)	0.065 (−0.34 to 0.45)
	Sdec	−0.124 (−0.50 to 0.29)	0.035 (−0.37 to 0.43)	−0.008 (−0.40 to 0.39)	0.126 (−0.28 to 0.50)	0.220 (−0.19 to 0.57)	−0.045 (−0.43 to 0.36)	0.241 (−0.17 to 0.58)	0.217 (−0.20 to 0.56)	0.092 (−0.32 to 0.47)
RSA 15 + 15	RSA_total_	−0.044 (−0.43 to 0.36)	0.186 (−0.23 to 0.54)	−0.047 (−0.44 to 0.35)	0.145 (−0.27 to 0.51)	−0.019 (−0.41 to 0.38)	0.090 (−0.32 to 0.47)	−0.054 (−0.44 to 0.35)	−0.095 (−0.47 to 0.31)	−0.161 (−0.52 to 0.25)
	RSA_best_	−0.016 (−0.41 to 0.38)	0.304 (−0.10 to 0.62)	0.087 (−0.32 to 0.47)	0.126 (−0.28 to 0.50)	−0.169 (−0.53 to 0.24)	0.111 (−0.30 to 0.49)	0.087 (−0.32 to 0.47)	−0.040 (−0.43 to 0.36)	−0.156 (−0.52 to 0.26)
	%Change	−0.011 (0.41 to 0.39)	0.123 (−0.29 to 0.49)	−0.127 (0.50 to 0.28)	0.152 (−0.26 to 0.52)	−0.324 (−0.64 to 0.08)	0.272 (−0.14 to 0.60)	−0.132 (−0.50 to 0.28)	0.038 (−0.36 to 0.43)	−0.052 (−0.44 to 0.35)
	Sdec	−0.035 (−0.42 to 0.37)	−0.221 (−0.57 to 0.19)	−0.202 (−0.55 to 0.21)	−0.004 (−0.40 to 0.39)	0.235 (−0.18 to 0.58)	−0.066 (−0.45 to 0.34)	−0.212 (−0.56 to 0.29)	−0.078 (−0.46 to 0.33)	0.018 (−0.38 to 0.41)
RSA-Curve	RSA_total_	−0.058 (−0.44 to 0.35)	0.159 (−0.25 to 0.52)	−0.189 (−0.54 to 0.22)	0.169 (−0.24 to 0.53)	0.115 (−0.29 to 0.49)	−0.031 (−0.42 to 0.37)	0.103 (−0.30 to 0.48)	0.068 (−0.34 to 0.45)	0.173 (0.24 to 0.53)
	RSA_best_	0.001 (−0.39 to 0.40)	0.175 (−0.24 to 0.53)	−0.196 (−0.55 to 0.22)	−0.022 (−0.41 to 0.38)	−0.022 (−0.41 to 0.38)	0.011 (−0.39 to 0.40)	−0.086 (−0.47 to 0.32)	0.042 (−0.43 to 0.36)	−0.042 (−0.43 to 0.36)
	%Change	0.155 (−0.26 to 0.52)	−0.011 (−0.41 to 0.39)	0.387 (−0.01 to 0.68)	0.329 (−0.08 to 0.64)	0.329 (−0.08 to 0.64)	0.168 (−0.24 to 0.53)	0.625* (0.31 to 0.82)	0.293 (−0.12 to 0.62)	0.285 (−0.12 to 0.61)
	Sdec	−0.123 (−0.49 to 0.29)	0.002 (−0.39 to 0.40)	−0.006 (−0.40 to 0.39)	0.361 (−0.04 to 0.66)	0.361 (−0.08 to 0.64)	−0.092 (−0.47 to 0.31)	0.353 (−0.05 to 0.66)	0.208 (−0.20 to 0.56)	0.388 (−0.01 to 0.68)

RSA 20 + 20 = 6 sets of 20+20 m with a rest of 20 s; RSA-Linear = 8 sets of 30 m with a rest of 25 s; RSA-RANDOM = 6 sets of 20 m (10+10 m, COD) with a rest of 20 s; RSA 15 + 15 = 5 x 30-m shuttle sprints (15 m + 15 m) with a rest of 30 s; RSA-Curve = 6 x 17-m (3 for each side) curvilinear sprints with a rest of 20 s; RSA_total_ = total time to cover all the sprints; RSA_best_ = the best time obtained in a single sprint; %Change = percentage of change between the first and the last sprint; Sdec = fatigue index; 5 m = sprint time at the 5^th^ m; 10 m = sprint time at the 10^th^ m; 30 m = sprint time at the 30^th^ m; CV_Best = fastest side in the curve sprint test; CV_Worst = slowest side in the curve sprint test; COD = change of direction test; SJ = squat jump; CMJ = countermovement jump; DJ = drop jump. ^⁎^ p < 0.05

## Discussion

The aim of this research was to establish the relationship between performance in different RSA tests in elite youth soccer players. The main results indicated moderate relationships between the RSA-RANDOM test and the RSA 20 + 20 test in the RSA_total_. However, for the same variable (RSA_total_), the RSA-Curve test showed a negative relationship with the RSA-Linear test, the RSA-RANDOM test and the RSA 15 + 15 test. In terms of RSA_best_, a moderate relationship was observed between the RSA 15 + 15 and the RSA-Linear test. Regarding the relationships between the RSA tests and the fitness tests, the results indicated moderate relationships between the RSA-Linear test (RSA_best_) and the 10-m sprint, and between the RSA-RANDOM test (RSA_total_ and RSA_best_) and the COD test. In addition, the RSA-Linear test (%Change) presented a moderate relationship with the CMJ, CV_Best and CV_Worst. Finally, negative relationships were found between the RSA 20 + 20 test (RSA_best_) and CV_Worst.

In terms of RSA_total_, our results showed a moderate relationship between the RSA-RANDOM and RSA 20 + 20 tests (Table 1). One of the similar characteristics of these two tests is recovery time (20 s). Although total recovery will be determined by the time available to perform this task, recovery after maximal effort (i.e., sprint) is mainly associated with phosphocreatine resynthesis (PCr), lactate metabolism and the elimination of intracellular inorganic phosphate (Glaister et al., 2005). Another aspect to be assessed is the type of recovery (active vs. passive). In this line, it has been shown that the type of recovery did not reveal differences in lactate concentration at the end of the RSA test (Castagna et al., 2008). Another aspect that both tests have in common is the number of sprints performed. Performance in an RSA test depends, in the first repetitions, on the utilization of high-energy phosphates (ATP and PCr), but in the last repetitions, it is aerobic capacity that determines performance (Thébault et al., 2011). In addition, both tests include a COD, and although they are of different angles (45º vs. 180º) and require different mechanical capabilities, the ability to change direction is present in both. It is true that the RSA-RANDOM test necessitates not only mechanical and neuromuscular skills, but also cognitive abilities (decision-making), potentially leading to a less favourable relationship between the two tests. Furthermore, considering the same variable (RSA_total_), the present results indicate a negative relationship between the RSA-Curve test and the others (Table 1). Biomechanical and neuromuscular differences of the curved sprint compared to the linear one (Filter et al., 2020b) may show a negative relationship with the different RSA tests used in this study. Furthermore, the RSA-Curve test is not a standard RSA test, but an adaptation of a curvilinear test (Filter et al., 2020b). Future research will be necessary to investigate the potential impact of biomechanical and neuromuscular differences in curved sprinting on RSA performance in soccer players.

In terms of RSA_best_, a moderate relationship was observed between the RSA-Linear and RSA 15 + 15 tests (Table 1). Although the first test is linear and the other consists of shuttle sprints, the distance covered is the same (30 m). Our results were in line with those found by Wong et al. (2012), who related an RSA test to an RCOD (repeated change of direction) test. Those authors found a correlation between the best sprint performance in the RSA test and the best sprint performance in the RCOD test. Therefore, soccer players with a good sprint performance would also have a good COD sprint performance (Wong et al., 2012). To conclude, there were no significant differences between these RSA tests considering the rest of the analysed variables, which shows a different nature of the two tests. Consequently, while both tests’ primary goal is to measure RSA, other implications of their configuration must be considered when selecting tests.

Regarding the relationships between the RSA and the fitness tests, the results indicated that the RSA-Linear test (RSA_best_) had a moderate relationship with the 10-m sprint performance (Table 2). The RSA-Linear test was the only one where a 30-m sprint was performed without braking, acceleration or changing the direction. As demonstrated, horizontal propulsive forces are of vital importance in the early acceleration phase of the sprint (Rabita et al., 2015), which has a positive impact on the further development of speed (Behrens and Simonson, 2011). As a result, the ability to accelerate can have a great influence on the best time in any linear RSA test. In addition, the RSA-RANDOM test (RSA_best_ and RSA_total_) showed moderate relationships with the COD test (Table 2). During the performance of this test, decision making is carried out prior to changing direction. The inclusion of this type of action in this study’s training sessions (i.e., exercises with decision making) could favour the improvement in the athletes' COD (Gonzalo-Skok et al., 2023). In addition, the %Change in the RSA-Linear test also had a moderate relationship with CV_Best, CV_Worst and the CMJ (Table 2). These results are in line with previous research, where a relationship between RSA performance and vertical jump height was observed (Slimani and Nikolaidis, 2019). Finally, the results showed a negative relationship between the RSA 20 + 20 test (RSA_best_) and CV_Worst. These findings are related to those discussed above, where the poor relationship between the RSA-Curve and the other RSA tests was observed.

There are certain limitations of this study that should be acknowledged: (1) the training load was not taken into account, which could influence the performance in the tests, although a 48-hour rest period between evaluation sessions was always respected; (2) although the number of participants in this study was similar to other research in this field, sample size was somewhat limited. Therefore, future studies are needed to obtain more evidence on the relationship between RSA tests with different characteristics (i.e., distance, sets, recovery time, linear or with change of direction, passive or active recovery, with decision-making or without etc.), as well as the relationship of RSA with other important physical qualities in the performance of soccer players, such as jumping, sprinting or changing direction. Furthermore, bearing in mind the existing sex differences (Tortu et al., 2024) when applying RSA protocols and differences in the maturational status (Perroni et al., 2024), it would be interesting to replicate these results taking into account these variables.

## Conclusions

In conclusion, when relating the performance between the different tests, it was observed that players who performed well in the RSA-RANDOM test also performed well in the RSA 20 + 20 test, and those who performed well in the RSA 15 + 15 test also performed well in the RSA-Linear test. In addition, negative relationships were observed between the RSA-Curve test and the other RSA tests. Therefore, the choice of the test to assess the RSA can be a function of the variable (RSA_best_ or RSA_total_), taking into account that the RSA-Curve test should not be used because of its negative relationship with the other RSA tests. Finally, when relating RSA tests to physical qualities, the 10-m linear sprint, CV_Best, CV_Worst and the CMJ were shown to have a good relationship with the RSA-Linear test, while the COD had a good relationship with the RSA-RANDOM test. As a result, improving these variables could have a positive influence on RSA performance. Due to the limited time available for RSA training, these findings could be valuable for coaches and sports scientists seeking to optimize training protocols and evaluate athlete performance more effectively. In this regard, based on these results, the good relationship between different RSA tests would help physical trainers to choose which test is better depending on the variable to be evaluated. In addition, training focused on improving different physical qualities (i.e., jumping, sprinting, changing direction etc.) can positively impact RSA performance. Therefore, soccer academies for youth players should focus their objectives on optimizing the different performance variables, taking into account what training means and which methods they can use knowing that there are physical qualities with a positive and others with a negative relationship.
